# Supplementary material for: Image-based high-throughput mapping of TGF-β-induced phosphocomplexes at a single-cell level
Source: Commun Biol. 2021 Nov 12;4:1284. doi: 10.1038/s42003-021-02798-4 (PMC8590043; doi:10.1038/s42003-021-02798-4)
Supplement: Supplementary file 2 — Supplementary Information [file 42003_2021_2798_MOESM2_ESM.pdf]

Supplementary information document for:

Image-based high-throughput mapping of TGF-β induced phosphocomplexes at a single cell level

Peter Lönn[1,#,*], Rasel A. Al-Amin[1,#], Ehsan Manouchehri Doulabi[1], Johan Heldin[1,2], Radiosa Gallini[1], Johan Björkesten[1], Johan Oelrich[1], Masood Kamali-Moghaddam[1], Ulf Landegren[1*]

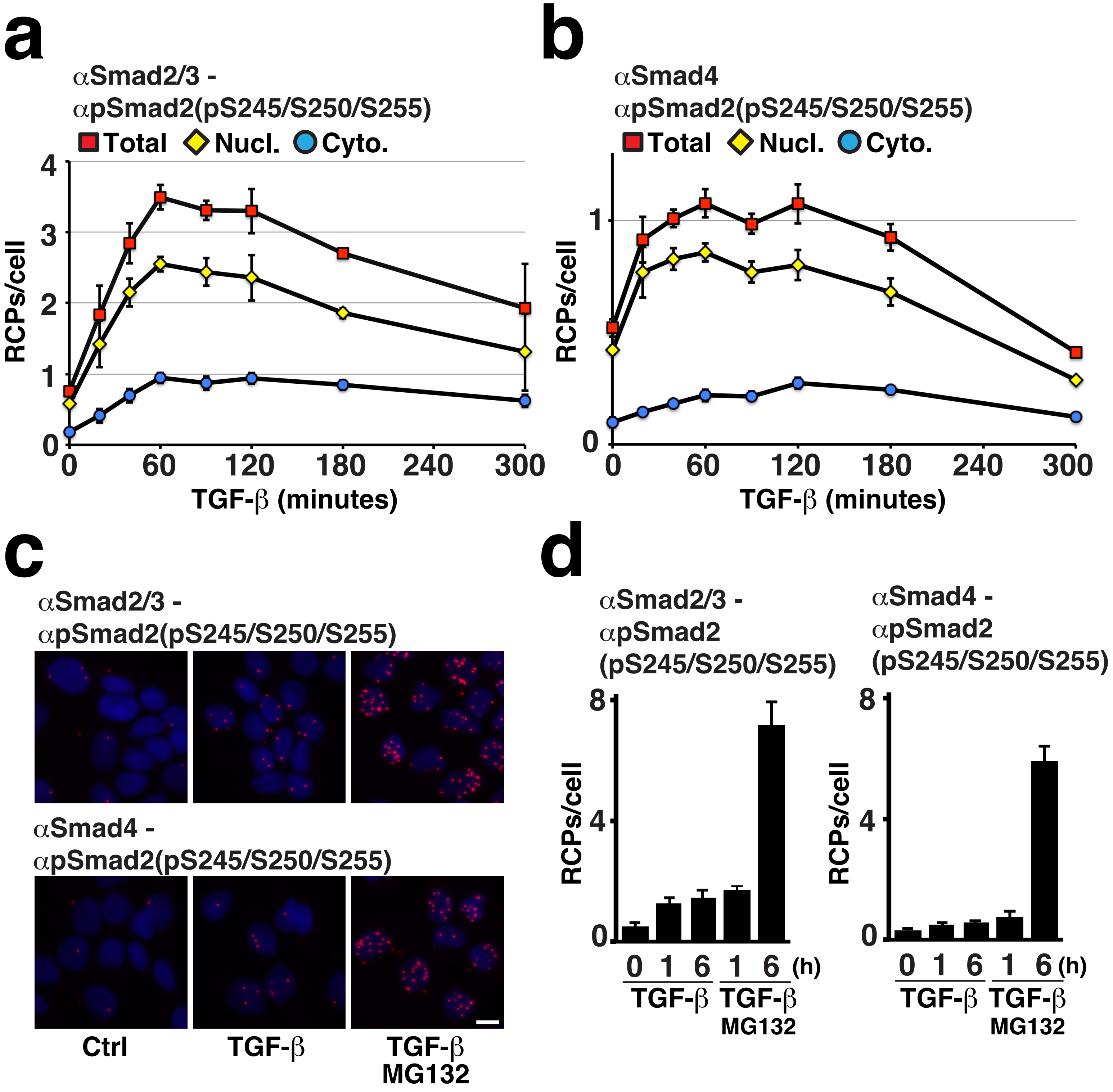

Supplemental Figure 1. Smad2(pS245/pS250/pS255) linker phosphorylation of starved HaCAT cells in response to 10 ng/ml TGF-β was visualized by high-throughput isPLA. (a, b) Time courses for the accumulation of RCPs representing complexes of Smad2/3-Smad2(pS245/pS250/pS255) and Smad4-Smad2(pS245/pS250/pS255), respectively. The red squares indicate average RCPs/cell, yellow diamonds indicate average RCPs/cell that overlap with the nuclear stain, and blue circles indicate average RCPs/cell that do not overlap with the nuclear stain. Results are displayed as mean RCPs/cell from triplicate wells (n=3), with nine images acquired and analyzed per well, and with standard deviations (SD) of the three analyzed wells displayed. (c) Fluorescence microscopy images of starved HaCAT cells and of cells treated with 10 ng/ml TGF-β for 6 h with or without the proteasomal inhibitor MG132. The scale-bars represent 10 μm. (d) HaCAT cells were starved and treated with 10 ng/ml TGF-β with or without MG132 for the indicated times and nine images (n=9) were acquired and analyzed per well. Bar graphs shows mean RCPs/cell with SD of the nine analyzed images. Data is shown as the average of two repeated wells.

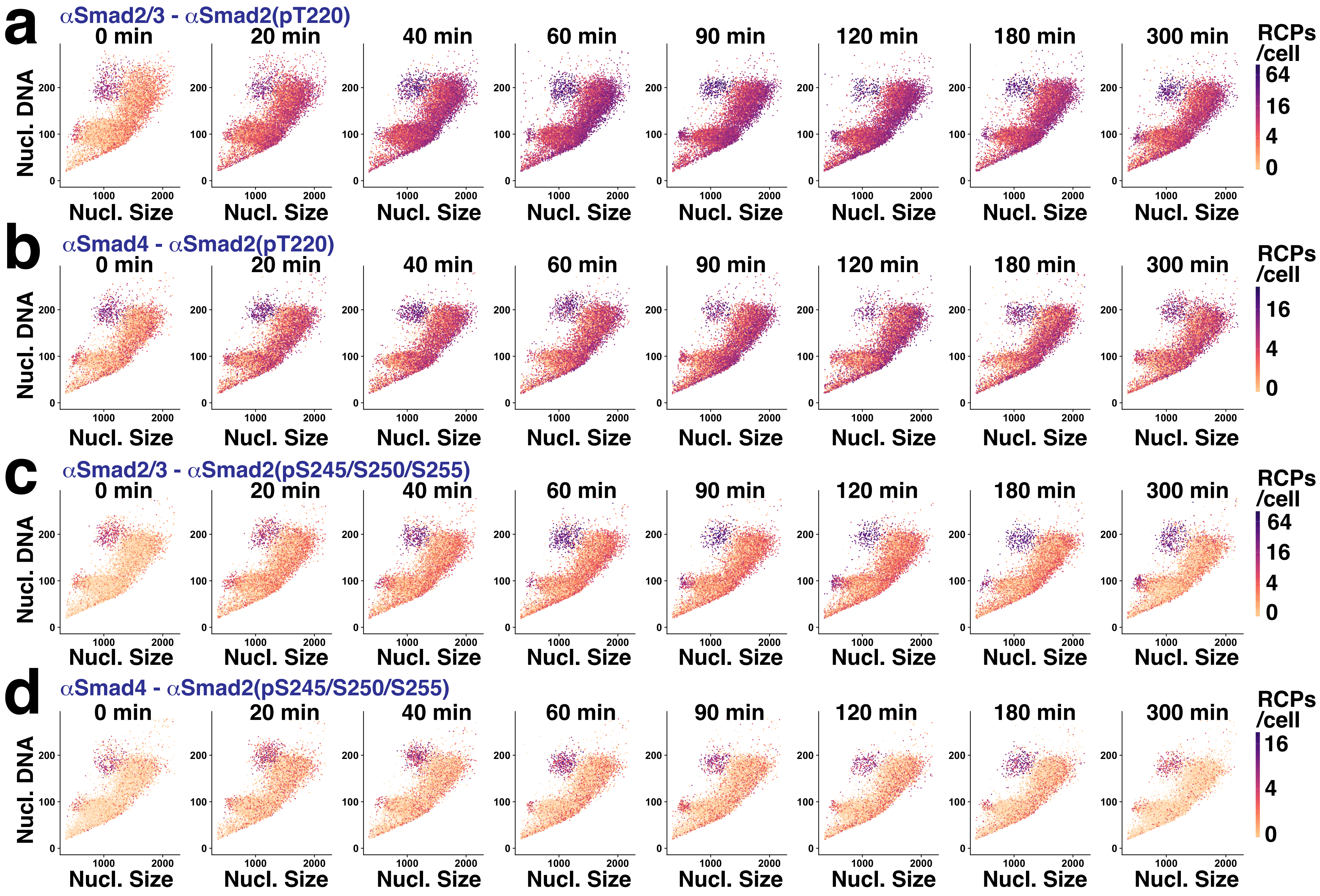
a
αSmad2/3 - αSmad2(pT220)
b
αSmad4 - αSmad2(pT220)
c
αSmad2/3 - αSmad2(pS245/S250/S255)
d
αSmad4 - αSmad2(pS245/S250/S255)
0 min
20 min
40 min
60 min
90 min
120 min
180 min
300 min
Nucl. DNA
Nucl. Size
RCPs /cell
64
16
4
0

Supplemental Figure 2. Smad2 linker phosphorylations and complex formations in individual starved HaCAT cells as a function of time of stimulation with 10 ng/ml TGF-β. (a-d) Numbers of RCPs, representing the identified phosphoproteins and complexes for individual HaCAT cells are indicated by color scales. The cells are plotted according to their nuclear sizes along the X-axes (pixels^2 (~ 0.11 μm^2)) and the intensity of the nuclear DNA stain along the Y-axes. (a) Results for isPLA using antibodies against Smad2/3 and Smad2(pT220). (b) Results for antibodies against Smad4 and Smad2(pT220). (c) Results using antibodies against Smad2/3 and Smad2(pS245/pS250/pS255). (d) Results for antibodies directed against Smad4 and Smad2(pS245/pS250/pS255). The log2 color scales indicate 0 - 96 RCPs/cell for (a), (c), 0 - 32 RCPs/cell for (b), and 0 – 24 for (d).

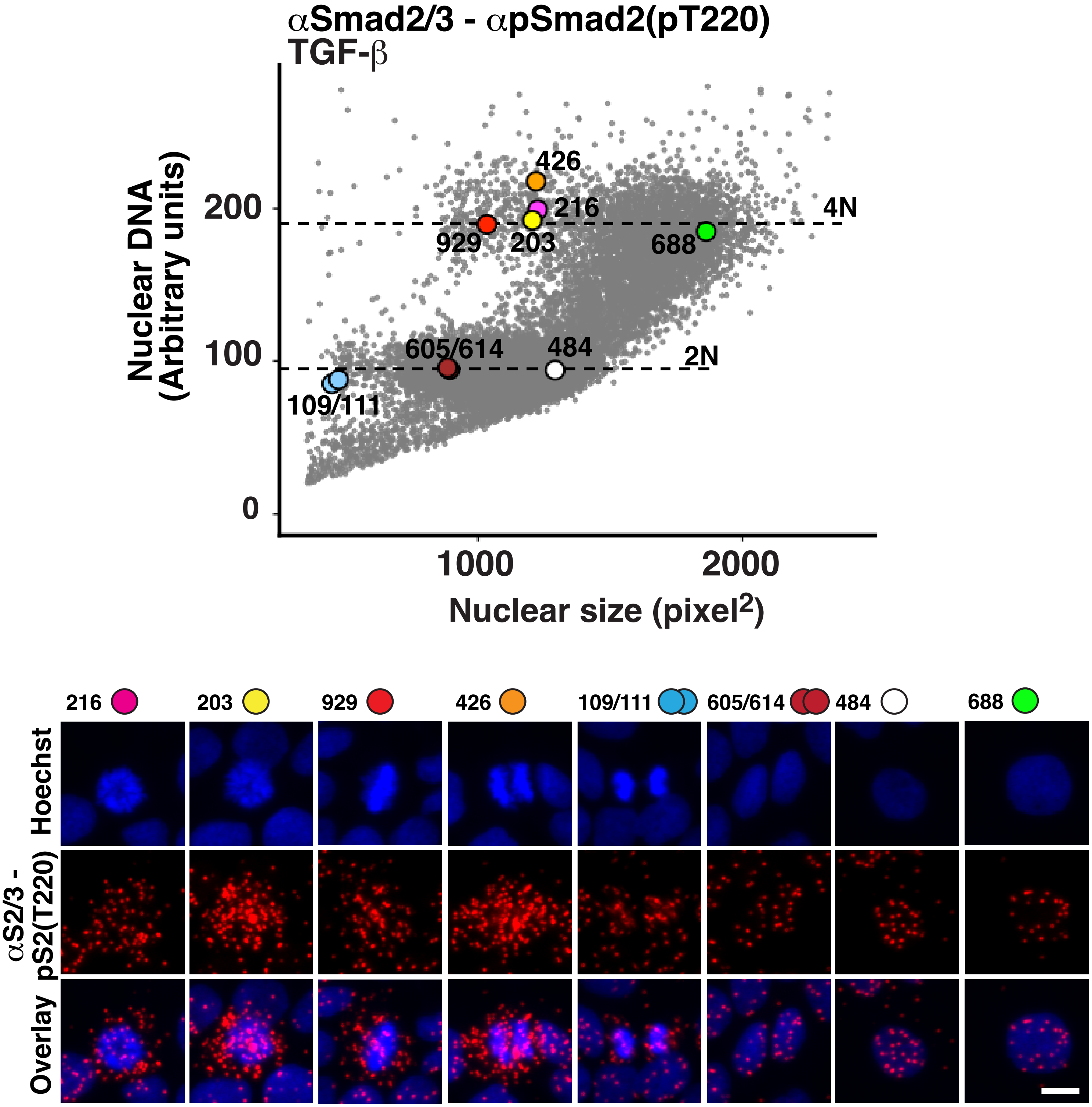

Supplemental Figure 3. Visualizing HaCAT nuclei in different phases of the cell-cycle. HaCAT nuclei were identified and displayed as Hoechst stained images (blue) overlayed with pSmad2(pT220) RCPs (red) (bottom panel) and the corresponding positions in the graphs displaying cells according to their nuclear stain vs nuclear size were highlighted (top panel). Color-coded circles together with an object number for each nucleus relates the images to the position of the cells in the graph.

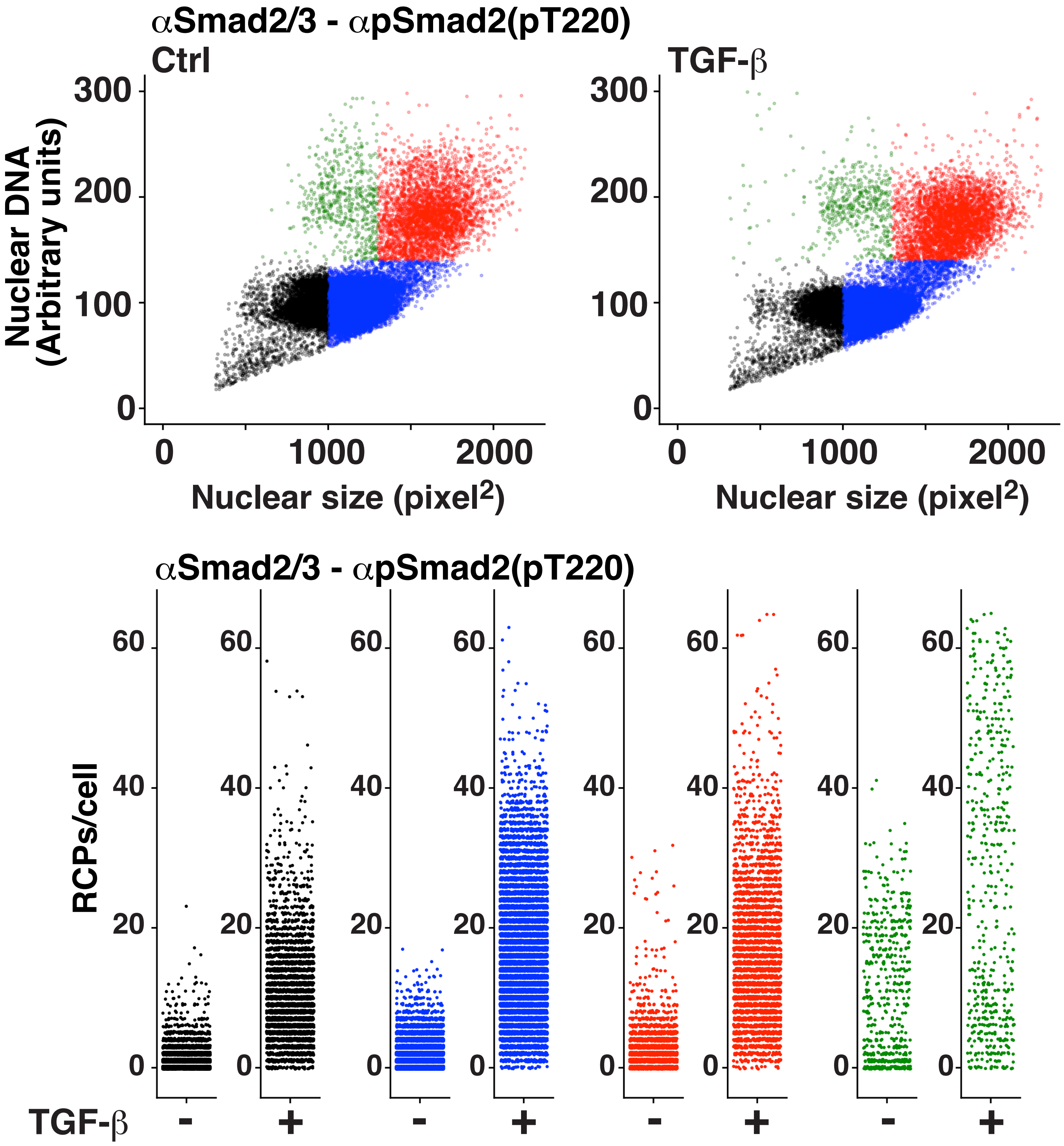

Supplemental Figure 4. Visualizing Smad2/3-Smad2(pT220) signaling in different phases of the cell-cycle. Individual HaCAT cells, starved and stimulated or not with TGF-β for 60 min, were distributed in the plots according to the nuclear area of each cell along the X-axes (pixels^2 (~ 0.11 μm^2)), and the integral intensity of the nuclear DNA stain along the Y-axes (Top panels). Cells were then identified and grouped based on their position in the graphs according to the displayed color code in the top panels (black, blue, red, and green). To visualize isPLA signals by cells in the different groups the unstimulated and stimulated RCPs per cell were plotted for each group in individual graphs using the same color coding as described above (bottom panels). The groups were segmented according to the following Y and X values: black (≤140, ≤1000), blue (≤140, >1000), red (>140, >1300), and green (>140, ≤1300).

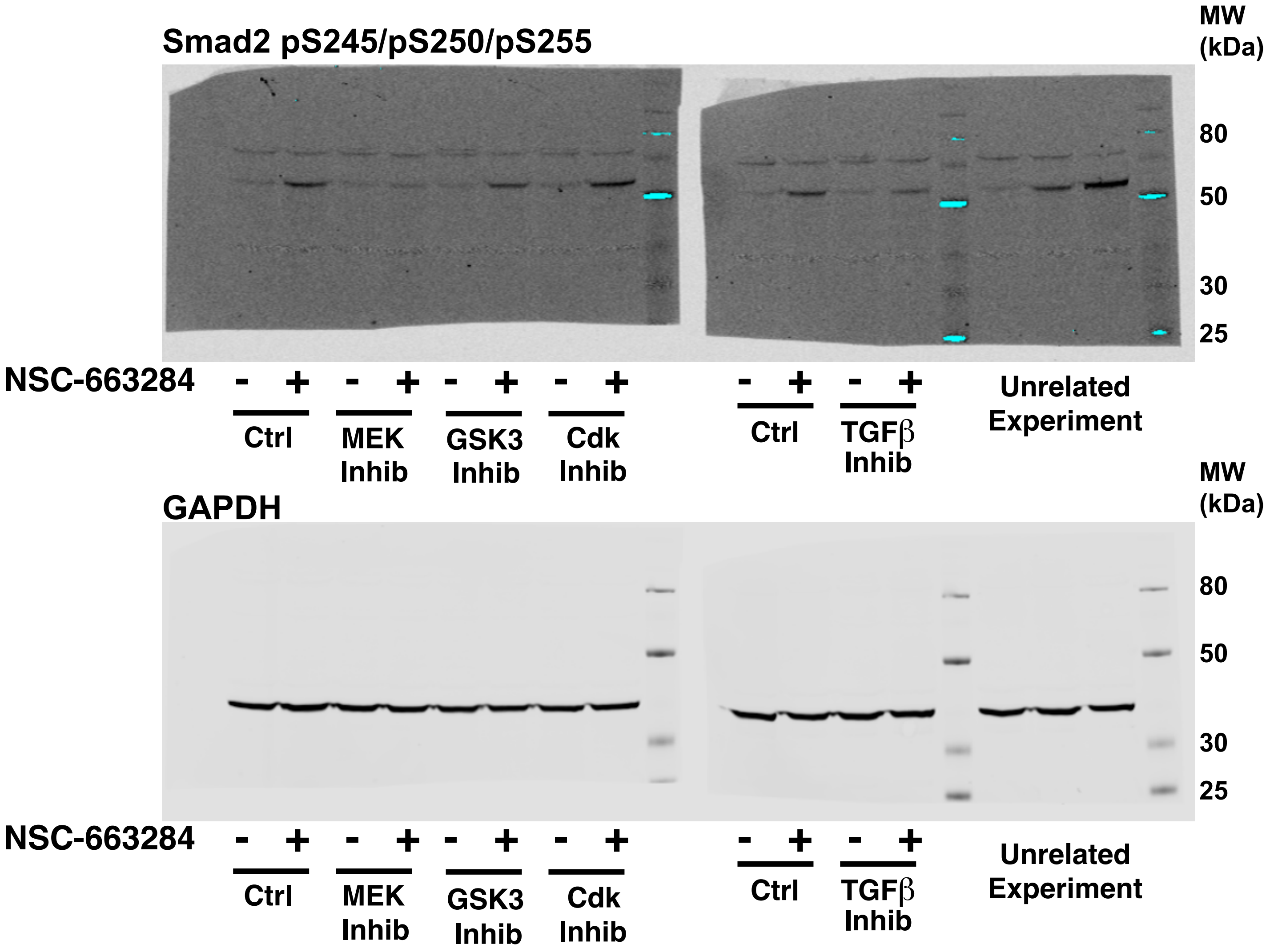

Supplemental Figure 5. Full western blot scans. Images are displaying the full western blot scans of Figure 5b with protein markers. Smad2(pS245/pS250/pS255) linker phosphorylation blots are shown in the top panel and GAPDH in the bottom panel.
