# Supplementary material for: Image-based high-throughput mapping of TGF-β-induced phosphocomplexes at a single-cell level
Source: Commun Biol. 2021 Nov 12;4:1284. doi: 10.1038/s42003-021-02798-4 (PMC8590043; doi:10.1038/s42003-021-02798-4)
Supplement: Supplementary file 3 — Description of Additional Supplementary Files [file 42003_2021_2798_MOESM3_ESM.pdf]

Description of Additional Supplementary Files

File name: Supplementary Data 1

Description: Source data for key figures.
