# Supplementary material for: Image-based high-throughput mapping of TGF-β-induced phosphocomplexes at a single-cell level
Source: Commun Biol. 2021 Nov 12;4:1284. doi: 10.1038/s42003-021-02798-4 (PMC8590043; doi:10.1038/s42003-021-02798-4)
Supplement: Supplementary file 5 — Reporting Summary [file 42003_2021_2798_MOESM5_ESM.pdf]

nature research

Corresponding author(s): Ulf Landegren and Peter Lönn

Last updated by author(s): Sep 22, 2021

Reporting Summary

Nature Research wishes to improve the reproducibility of the work that we publish. This form provides structure for consistency and transparency in reporting. For further information on Nature Research policies, see our Editorial Policies and the Editorial Policy Checklist.

Statistics

For all statistical analyses, confirm that the following items are present in the figure legend, table legend, main text, or Methods section.

n/a	Confirmed	
☐	☒	The exact sample size (*n*) for each experimental group/condition, given as a discrete number and unit of measurement
☐	☒	A statement on whether measurements were taken from distinct samples or whether the same sample was measured repeatedly
☒	☐	The statistical test(s) used AND whether they are one- or two-sided<br>*Only common tests should be described solely by name; describe more complex techniques in the Methods section.*
☒	☐	A description of all covariates tested
☒	☐	A description of any assumptions or corrections, such as tests of normality and adjustment for multiple comparisons
☐	☒	A full description of the statistical parameters including central tendency (e.g. means) or other basic estimates (e.g. regression coefficient) AND variation (e.g. standard deviation) or associated estimates of uncertainty (e.g. confidence intervals)
☒	☐	For null hypothesis testing, the test statistic (e.g. *F*, *t*, *r*) with confidence intervals, effect sizes, degrees of freedom and *P* value noted<br>*Give P values as exact values whenever suitable.*
☒	☐	For Bayesian analysis, information on the choice of priors and Markov chain Monte Carlo settings
☒	☐	For hierarchical and complex designs, identification of the appropriate level for tests and full reporting of outcomes
☒	☐	Estimates of effect sizes (e.g. Cohen's *d*, Pearson's *r*), indicating how they were calculated

Our web collection on statistics for biologists contains articles on many of the points above.

Software and code

Policy information about availability of computer code

Data collection	Cell images were aquired using a Molecular Devices ImageXpress micro XL automated microscopy and Molecular Devices Metaxpress software. CellProfiler version 3 was used to measure and analyze cell images (Cellprofiler.org). Western blots were aquired using a LI-COR Biosciences Odyssey Scanner and ImageStudio Lite system
Data analysis	Data analysis was perfomed on a MacBook Pro OS X (version 10.9.5) with R studio (version 1.1.423) to graph single cell data and Excel:mac (version 14.5.8).

For manuscripts utilizing custom algorithms or software that are central to the research but not yet described in published literature, software must be made available to editors and reviewers. We strongly encourage code deposition in a community repository (e.g. GitHub). See the Nature Research guidelines for submitting code & software for further information.

Data

Policy information about availability of data

All manuscripts must include a data availability statement. This statement should provide the following information, where applicable:

- Accession codes, unique identifiers, or web links for publicly available datasets
- A list of figures that have associated raw data
- A description of any restrictions on data availability

The authors declare that the main data supporting the key findings of this study are available within the article and its Supplementary files. Additional source data are available from the corresponding authors upon reasonable request.

Field-specific reporting

Please select the one below that is the best fit for your research. If you are not sure, read the appropriate sections before making your selection.

☒ Life sciences ☐ Behavioural & social sciences ☐ Ecological, evolutionary & environmental sciences

For a reference copy of the document with all sections, see nature.com/documents/nr-reporting-summary-flat.pdf

Life sciences study design

All studies must disclose on these points even when the disclosure is negative.

Sample size	isPLA Smad2 phosphorylation and interaction time-courses and single cell analyses (Figures 1B,C,D; 2A,B,C,D; 4A,B; Suppl. 1A,B ; Suppl. 2A,B,C,D; Suppl. 3A, Suppl. 4A,B) were performed in 96-well cell culture plates. Cells were grown in triplicate (3) wells for each condition and nine images were unbiasly collected and analyzed per well using automated microscopy. For the single cell data plots, all cells aquired in all images were analyzed and displayed.<br><br>isPLA Smad2 phosphorylation and interaction graphs after MG132 (Figure 1E, F) were performed in 96-well cell culture plates. Cells were grown in triplicate (3) wells for each condition and nine images were unbiasly collected and analyzed per well using automated microscopy.<br><br>isPLA Smad2 phosphorylation and interaction analyses after MG132 (Figure 3A,B,C,D, Suppl. Figure 1C,D) were performed in 96-well cell culture plates. Cells were grown in duplicate (2) wells for each condition and nine images were unbiasly collected and analyzed per well using automated microscopy. For the single cell data plots, all cells aquired in all images were analyzed and displayed.<br><br>isPLA Smad2 phosphatase screen (Figure 5A) were performed in 96-well cell culture plates. Cells were grown in duplicate (2) wells for each condition and nine images were unbiasly collected and analyzed per well using automated microscopy. The accompanying western blot (Figure 5B) only served as an orthogonal method replicate of the NSC-663284 screen result and was therefore only perfomed once for confirmation (6-well plates with one well per condition).
Data exclusions	No data has been excluded from the analysis.
Replication	Data from the semi-automated isPLA, aquired with automated microscopy and anlysed by computer-based image analysis, are displayed as representative datasets of experiments that has been performed at least twice. For the western blot image (Figure 5B), this assay only serves as an independent orthogonal method replicate to confirm the NSC-663284 isPLA screen results and has therfore only been perfomed in this specific setup once.
Randomization	No randomization studies has been performed as it has not been relevant for this in vitro study.
Blinding	Investigators were not blinded during experiments. However, by using automated microscopy and cell profiler automated analysis, the majority of the data in the paper is completely unbiasly (blindly) collected and analysed.

Reporting for specific materials, systems and methods

We require information from authors about some types of materials, experimental systems and methods used in many studies. Here, indicate whether each material, system or method listed is relevant to your study. If you are not sure if a list item applies to your research, read the appropriate section before selecting a response.

Materials & experimental systems

n/a	Involved in the study	
☐	☒	Antibodies
☐	☒	Eukaryotic cell lines
☒	☐	Palaeontology and archaeology
☒	☐	Animals and other organisms
☒	☐	Human research participants
☒	☐	Clinical data
☒	☐	Dual use research of concern

Methods

n/a	Involved in the study	
☒	☐	ChIP-seq
☒	☐	Flow cytometry
☒	☐	MRI-based neuroimaging

Antibodies

Antibodies used	Anti-Smad2(pS245/pS250/pS255) (#3104) was from Cell Signaling and anti-Smad2/3 (610842) from BD Bioscience. Anti-GAPDH (AM4300) was aquired from Life Technologies. Anti-Smad2(pT220) (sc-135644) and anti-Smad4 (sc-7966) were purchased from Santa Cruz Biotechnologies.
Validation	For isPLA, the assay uses pairs of antibodies for detection, meaning both a phospho-smad antibody and a Smad antibody must detect the protein in close proximity for signal. In addition to the suppliers details, the included experiments show that phospho-detection

only occurs after TGF-b stimulation, which is what would be expected and in line with litterature and therefore provides further assurance of the specificity

Eukaryotic cell lines

Policy information about cell lines

Cell line source(s)	HaCAT cells were a kind gift from the Ludwig Institue for Cancer Research, Uppsala.
Authentication	Cells have not been authenticated
Mycoplasma contamination	Cells in the lab are routinly tested for mycoplasma contaminations. Only cells that are tested negative are used.
Commonly misidentified lines (See ICLAC register)	No misidentified cell lines are used
